# Tool Wear and Surface Evaluation in Drilling Fly Ash Geopolymer Using HSS, HSS-Co, and HSS-TiN Cutting Tools

**DOI:** 10.3390/ma14071628

**Published:** 2021-03-26

**Authors:** Mohd Fathullah Ghazali, Mohd Mustafa Al Bakri Abdullah, Shayfull Zamree Abd Rahim, Joanna Gondro, Paweł Pietrusiewicz, Sebastian Garus, Tomasz Stachowiak, Andrei Victor Sandu, Muhammad Faheem Mohd Tahir, Mehmet Erdi Korkmaz, Mohamed Syazwan Osman

**Affiliations:** 1 Faculty of Mechanical Engineering Technology, Universiti Malaysia Perlis, Perlis 01000, Malaysia; shayfull@unimap.edu.my; 2 Center of Excellence Geopolymer and Green Technology (CEGeoGTech), Universiti Malaysia Perlis, Perlis 01000, Malaysia; sav@tuiasi.ro (A.V.S.); faheem@unimap.edu.my (M.F.M.T.); 3 Faculty of Chemical Engineering Technology, Universiti Malaysia Perlis, Perlis 01000, Malaysia; 4 Department of Physics, Częstochowa University of Technology, 42-200 Częstochowa, Poland; joanna.gondro@pcz.pl (J.G.); pawel.pietrusiewicz@pcz.pl (P.P.); 5 Faculty of Mechanical Engineering and Computer Science, Częstochowa University of Technology, 42-200 Częstochowa, Poland; gari.sg@gmail.com (S.G.); stachowiak@ipp.pcz.pl (T.S.); 6 Faculty of Material Science and Engineering, Gheorghe Asachi Technical University of Iasi, 41 D. Mangeron St., 700050 Iasi, Romania; 7 National Institute for Research and Development for Environmental Protection INCDPM, 294 Splaiul Inde-pendentei, 060031 Bucharest, Romania; 8 Machine Engineering Department, Faculty of Engineering, Karabuk University, 78050 Karabuk, Turkey; merdikorkmaz@karabuk.edu.tr; 9 Faculty of Chemical Engineering, Universiti Teknologi MARA, Cawangan Pulau Pinang, 13500 Permatang Pauh, Malaysia; syazwan.osman@uitm.edu.my

**Keywords:** geopolymer drilling, geopolymer machining, green materials

## Abstract

This paper reports on the potential use of geopolymer in the drilling process, with respect to tool wear and surface roughness. The objectives of this research are to analyze the tool life of three different economy-grade drill bit uncoated; high-speed steel (HSS), HSS coated with TiN (HSS-TiN), and HSS-cobalt (HSS-Co) in the drilling of geopolymer and to investigate the effect of spindle speed towards the tool life and surface roughness. It was found that, based on the range of parameters set in this experiment, the spindle speed is directly proportional to the tool wear and inversely proportional to surface roughness. It was also observed that HSS-Co produced the lowest value of surface roughness compared to HSS-TiN and uncoated HSS and therefore is the most favorable tool to be used for drilling the material. For HSS, HSS coated with TiN, and HSS-Co, only the drilling with the spindle speed of 100 rpm was able to drill 15 holes without surpassing the maximum tool wear of 0.10 mm. HSS-Co exhibits the greatest tool life by showing the lowest value of flank wear and produce a better surface finish to the sample by a low value of surface roughness value (Ra). This finding explains that geopolymer is possible to be drilled, and therefore, ranges of cutting tools and parameters suggested can be a guideline for researchers and manufacturers to drill geopolymer for further applications.

## 1. Introduction

The use of environmentally friendly materials has been increasing across the globe because it helps reduce waste, minimize pollution, and save energy. Geopolymer, for instance, is one of the green materials and has been highly demanded in numerous applications, particularly in construction materials, to replace ordinary cement [1]. The interest in these materials has been increasing due to the manufacturing of this material that involves a very low rate of greenhouse gas emission [2,3]. Apart from that, geopolymer utilizes waste materials such as recycled concrete and unwanted fly ash. This material has interesting properties because it is very durable and stable at high temperature; thus, this property has been one of the reasons why geopolymer materials are now widely used in many sectors, especially in the engineering sector such as civil construction, automotive industry, and aviation sector [2,3,4,5].

Geopolymer is a type of inorganic polymer with an amorphous structure that polymerizes in a quick reaction of silica (Si)-alumina (Al) under alkaline conditions, creating a three-dimensional polymeric chain of Si-O-Al-O bonds [6,7]. Understanding that the existence of Si and Al can be used to easily produce geopolymer, this material is easily made by using a pre-shaped mold in which all required raw materials are mixed and left solidified at room temperature [6,7,8]. The final shape of the material highly depends on the mold’s shape and its final dimensional accuracy is still an issue. The need to manufacture geopolymer in various forms and shapes with high accuracy is required so that its advantages can be widely benefited not just in construction applications. From the manufacturing point of view, one of the ways of producing materials in various forms and shapes can be achieved through machining processes [8].

Machining such as the drilling process is one of the important processes in shaping certain materials. Computer Numerical Control (CNC) milling machines, hole drilling apparatus, and milling cutters are used especially for drilling blind holes of certain depths that are not lengthwise and axial holes of certain precise dimensions. Basically, high-speed steel (HSS) drills are used in these processes. However, coated carbide and indexable drills have also been recently used [6,7,8,9,10,11].

Chip formation during the drilling process affects the cutting forces, cutting temperature, surface quality, and dimensional accuracy of the hole [6]. In addition, the disposability of the chip during the drilling operations directly affects the hole quality and changes according to the cutting parameters (spindle speed, feed). Amongst those parameters mentioned, spindle speed and feed are the most important parameters in drilling. These parameters directly affect cutting conditions that occur during the cutting process and are the factors that determine the performance of the cutting tool (drill) [11,12,13,14,15].

In the light of the studies in the literature, tool life (based on tool wear) and surface quality are therefore the main focus of this paper. Failure to control the flank wear occurring during the drilling process causes both the cutting tool and the workpiece to be significantly affected. It causes different types of wear on the cutting tool, eventually causing the tool to complete its life in a shorter time than expected. On the other hand, the surface quality of the workpiece is negatively affected, in addition to undesirable changes in the chemical structure of the workpiece. For these reasons, tool wear and surface roughness measurements in hole drilling have attracted the attention of many researchers.

Previous reports showed that fly ash geopolymer can be machined using milling and turning processes but with very low spindle speeds and small depth of cuts [16,17,18] but have yet to provide evidence on drilling performance on geopolymer materials. Apart from the articles reported, literature related to geopolymer drilling is still lacking, and in order to fill in the gap in the literature, this paper assessed the relationship between spindle speeds and tool life of drilling fly ash geopolymer using three types of cutting tools—HSS, HSS coated with TiN, and HSS-cobalt (HSS-Co).

## 2. Methodology

A set of class C fly ash geopolymer blocks was prepared with a size of 100 mm × 100 mm × 100 mm. The methods of preparing the samples were as previously reported [16,17,18]. Table 1 shows the list of apparatus used in producing the geopolymer samples.

The molarity of sodium hydroxide was controlled at 12 M whereby the ratio of sodium silicate (Na_2_SiO_3_) to sodium hydroxide (NaOH) was set at 2.5, and the solid-to-liquid ratio was set at 2.0, as employed in a previous report [19]. The calculation for determining the weight of fly ash and alkaline activator based on the cubic mold is as follows [18,19]:

- Sample size = 100 mm × 100 mm × 100 mm;
- Total weight of fly ash used = 1600 g;
- Solid-to-liquid ratio = 2.0.

The weight of the alkaline activator was obtained as follows:

(1) $$\frac{\;\mathrm{Weight}\;\mathrm{of}\;\mathrm{Fly}\;\mathrm{ash}}{\mathrm{Weight}\;\mathrm{of}\;\mathrm{Alkaline}\;\mathrm{activator}}=\frac{2.0}{1.0}$$

$$=\frac{1600\;g}{\mathrm{Weight}\;\mathrm{of}\;\mathrm{Alkaline}\;\mathrm{Activator}}=\frac{2.0}{1.0}$$

Weight of Alkaline Activator=800 g

Thus, the weight of NaOH + Na_2_SiO_3_ was 800 g. Since Na_2_SiO_3_/NaOH ratio was 2.5, the weight of Na_2_SiO_3_ was calculated as shown below.

(2) $$\frac{\mathrm{Weight}\;\mathrm{of}\;{\mathrm{Na}}_{2}{\mathrm{SiO}}_{3}}{\mathrm{Weight}\;\mathrm{of}\;\mathrm{NaOH}}=\frac{2.5}{1.0}$$

$$\mathrm{Weight}\;\mathrm{of}\;{\mathrm{Na}}_{2}{\mathrm{SiO}}_{3}=\frac{2.5}{2.5+1.0}\times 800\;g=\;571.429\;g$$

Weight of NaOH=800 g−571.429 g=228.571 g

**Figure 1:**
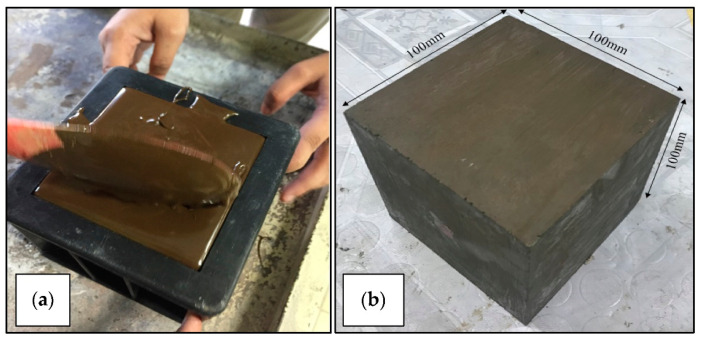
(**a**) Preparation process of geopolymer samples and (**b**) workpiece of fly ash geopolymer after solidified ready for drilling experiment.

Figure 1 shows the geopolymer block sample after solidification and ready for the drilling process.

**Figure 2:**
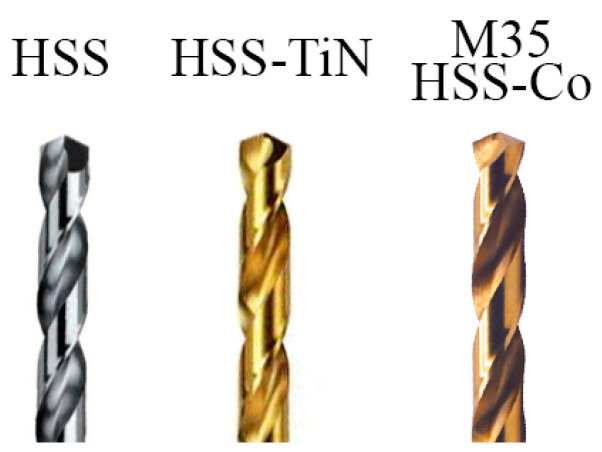
Images of high-speed steel (HSS), HSS coated with TiN (HSS-TiN), and M35 HSS-cobalt (HSS-Co) with new conditions.

**Figure 3:**
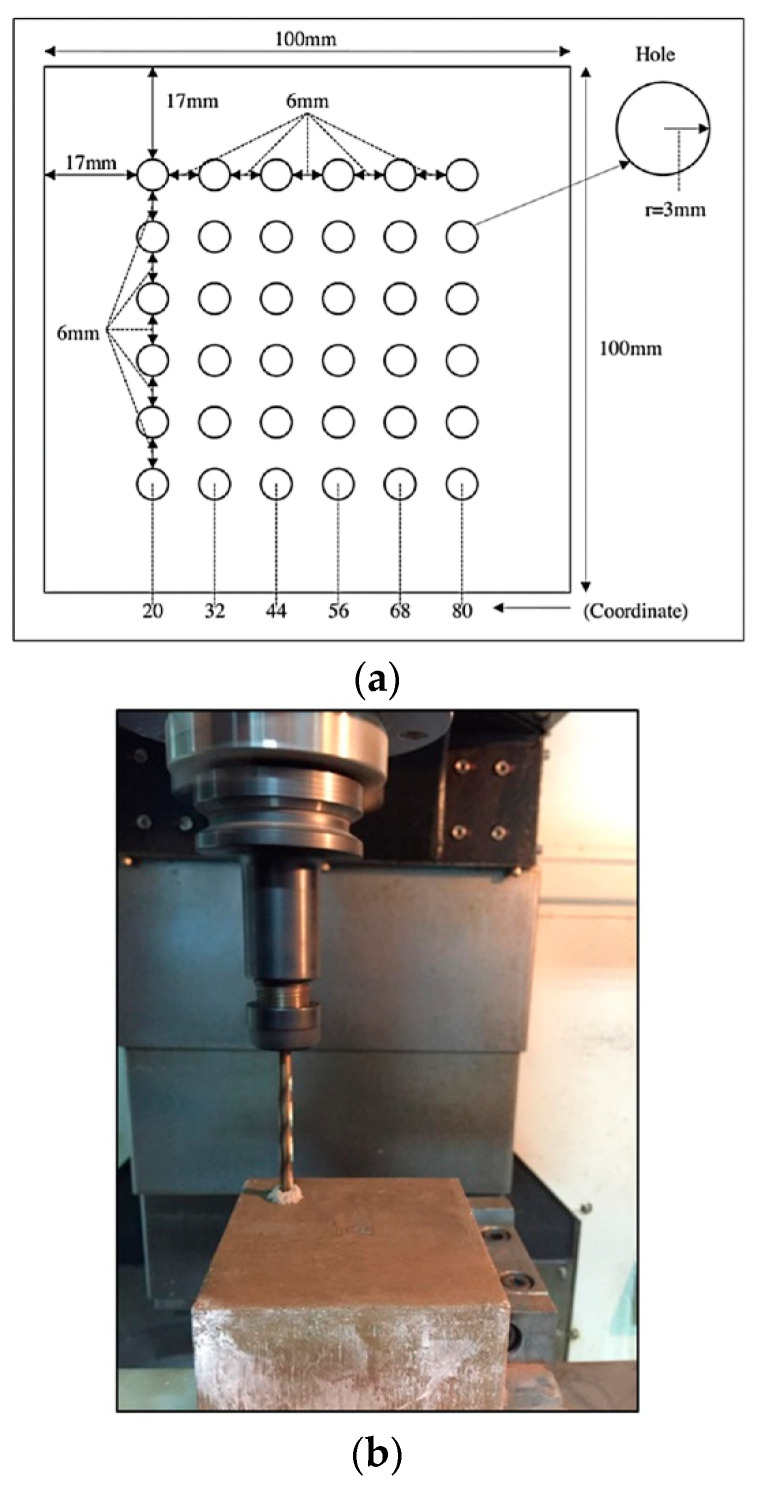
(**a**) Diagram of designated drilled holes on the workpiece and (**b**) drilling operation on geopolymer samples.

Three types of cutting tools—uncoated HSS, HSS-TiN, and HSS-Co—were selected for this experiment. HSS was chosen in this exploration study because this drill bit is affordable at a low price, and considering that it offers several advantages such as being easy to maintain with a seamless sharpening of blunt tools and having a high level of compactness and high breaking strength. HSS coated with TiN and HSS-Co were chosen as another set of samples because the coatings of TiN and the addition of cobalt elements in HSS were reported to offer more strength and increases durability [20]. The details of the drilling parameters are shown in Table 2, and the image of the cutting tools is shown in Figure 2. Six rows of 6.0-mm-hole diameter with 6.0-mm spaces in between each hole were set for drilling, as shown in Figure 3. The maximum tool wear (Vbmax) of 0.10 mm was set as the limit of the tool life as recommended by a previous report [21].

**Figure 4:**
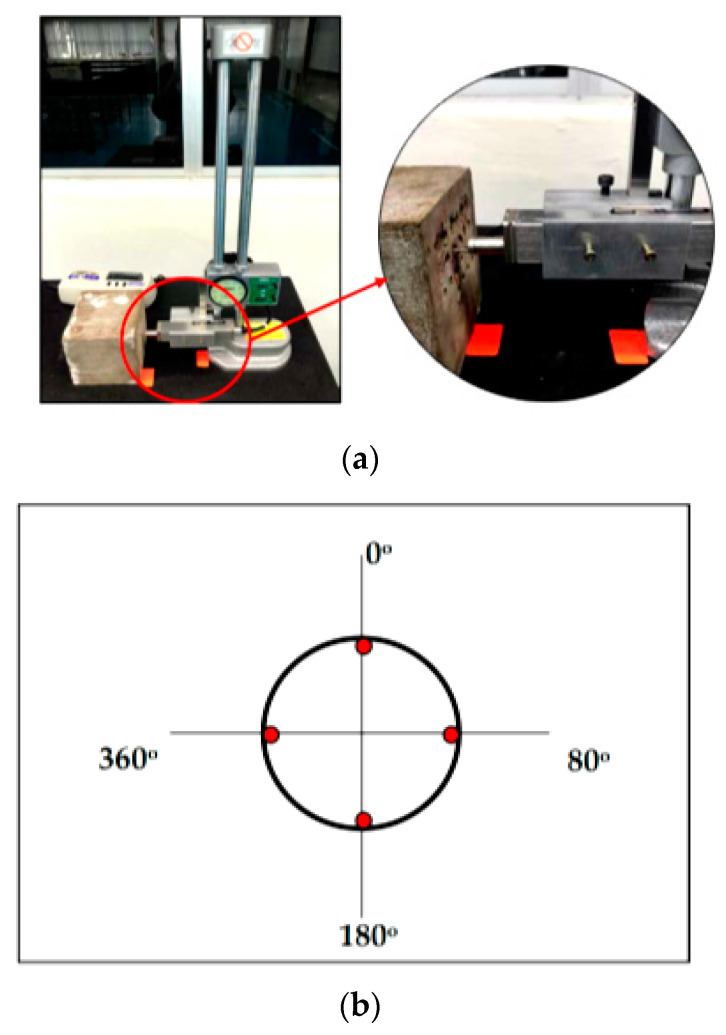
(**a**) The location of surface roughness measurement and (**b**) location of measurement.

Four different spindle speeds that were 250 rpm, 200 rpm, 150 rpm, and 100 rpm with a constant feed rate of 0.10 mm/rev and depth of cut 0.30 mm were set using Akira Seiki Performa SR3 CNC milling machine, as shown in Figure 4. Initially, the parameter selection was set in larger ranges based on the literature [22] with respect to the nearest similar samples; however, the samples experienced major cracks when drilled at various high spindle speed parameters, and therefore, the ranges had to be lowered until the drilling process could be completed highest at 250 rpm. The flank wear was observed in the sequence of 5, 10, 15, 20, and 25 holes using Xoptron Stereo Microscope XST60 digital microscope and visualized using iSolution Lite. The surface roughness was measured using Accretech Handysurf E^+^ 35 Portable Surface Tester on each sample. The setup of the surface roughness measurement is shown in Figure 4a, while the location of measurement is represented in Figure 4b.

## 3. Results and Discussion

### 3.1. Tool Wear Evaluation

**Figure 5:**
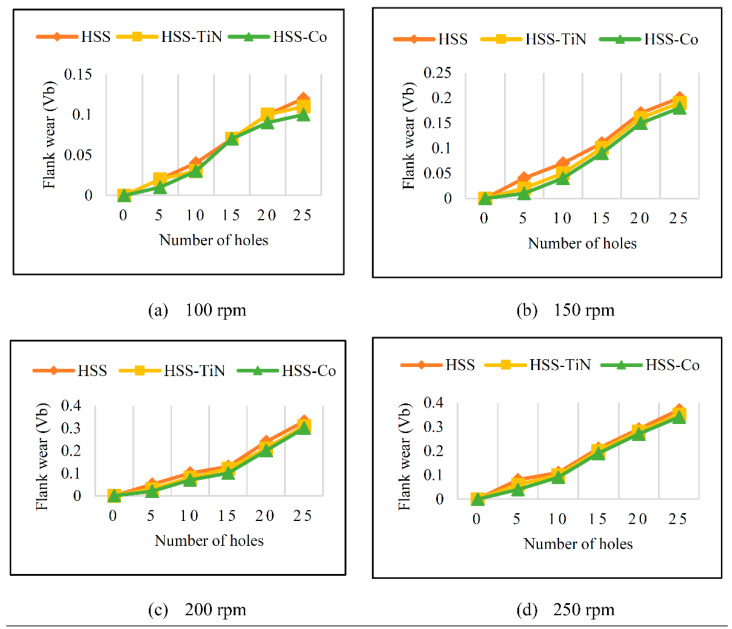
Growth of flank wear of HSS, HSS-TiN, and HSS-Co from (**a**–**d**).

Before discussing the flank wear, it is interesting to note that the chip formed observed is powder-like chips. This type of formed chip was due to the properties of the fly ash geopolymer, which is very hard and brittle [22]. Due to its brittleness, the geopolymer experience brittle fractures, therefore producing discontinuous chips and most of the time powder forms [23]. This powder-like chip also shows a sign of a potential reduction of the life of the bit, and even breaks the tool if high spindle speed is applied. Table 3 shows tool wear (flank wear) as measured from the optical microscope on different spindle speeds using HSS, HSS-TiN, and HSS-Co. The data were then tabulated into graphs, as shown in Figure 5. As expected, it can be observed that the flank wear increased as the drilling processes went through until Hole-25. Continuous employment of the drilling process from one hole to another is believed to have increased the cutting temperature, which had a huge impact on wear, experiencing an increase in the flank wear, as mentioned by the literature [24]. The images of the flank wear captured by the microscope for each cut are shown in Table 4. From the table, there is an existence of growing tool wear occurred due to the friction between the underside of the tool (clearance face) and the geopolymer workpiece. The wear happened at the drill corner due to subjected horizontal and vertical forces that occurred and from the spindle speed and contact length at the maximum. This is due to the higher friction that happened during the drilling process, contributing to the removal of small particles from the sharp edges of the cutting tools [25]. The cutting edges of the flank surface gradually wore as the drilling process progressed with no sign of flaking or chipping. Another interesting finding was that in the coating, the presence of Co grain started to wear slowly after a few holes until the end of hole 25, indicating that the coating is not significant in resisting flank wear. Previous research stated that HSS-Co has poor heat and wear resistance [26,27], and the corner edge starts to wear after the coating grains are lost in the worn zone. This justifies that both HSS-Co and HSS-TiN are the cheapest types of coated HSS in the market. However, both coated tools still manifest a better performance, compared to uncoated HSS.

**Figure 6:**
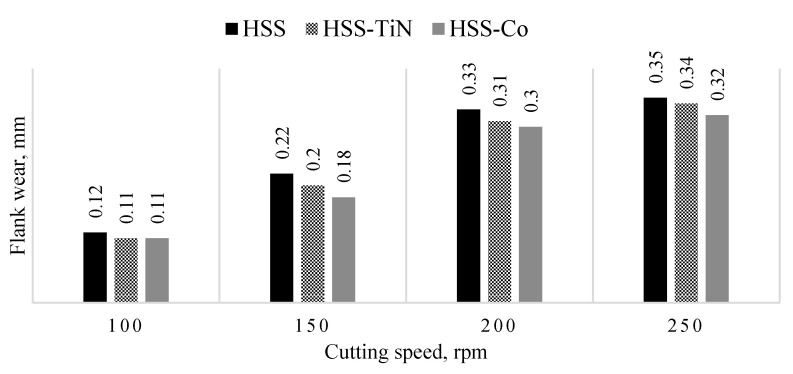
Flank wear value of cutting tools 25th hole at different spindle speeds.

Figure 6 shows the progression of flank wear curves for uncoated HSS, HSS-TiN, and HSS-Co drill bits in drilling the fly ash geopolymer at spindle speeds of 100, 150, 200, and 250 rpm at 25th holes. Although the major trends show that all flank wears increase as the spindle speed increases, it is interesting to note that at 100 rpm, the flank wear does not show significant differences. This result is in line with the previous studies that showed the increasing value of tool wear as spindle speed increases [22,26,28,29]. However, starting at 150 rpm, the flank wear differences show noteworthy differences. The presence of a layer of coating increases the hardness and wear resistance of the HSS-TiN and HSS-Co drill bits at low speed, and therefore, less wear occurred, compared to the uncoated HSS drill bits [26], but yet the coatings could not sustain the friction at 150 rpm. The highest flank wear was recorded on an uncoated HSS drill bit at 250 rpm, with a value of 0.35 mm. This highest is in line with the literature [26,27,28,29,30] due to the fact that HSS is less superior, compared to HSS-TiN coated and HSS-Co. The loss of the tool material can be observed on the tool flank, due to abrasive wear of the cutting edge against the machined surface. Although the tool material loss happened in all three cutting tools, the worst came from the uncoated HSS due to its direct engagement between the HSS materials and the workpiece. Based on the range of parameters used in this experiment, it demonstrates that spindle speed is directly proportional to the flank wear value. Increases in spindle speed reduce the tool-to-chip contact area, therefore reducing friction, which allows better surface quality to be achieved.

**Figure 7:**
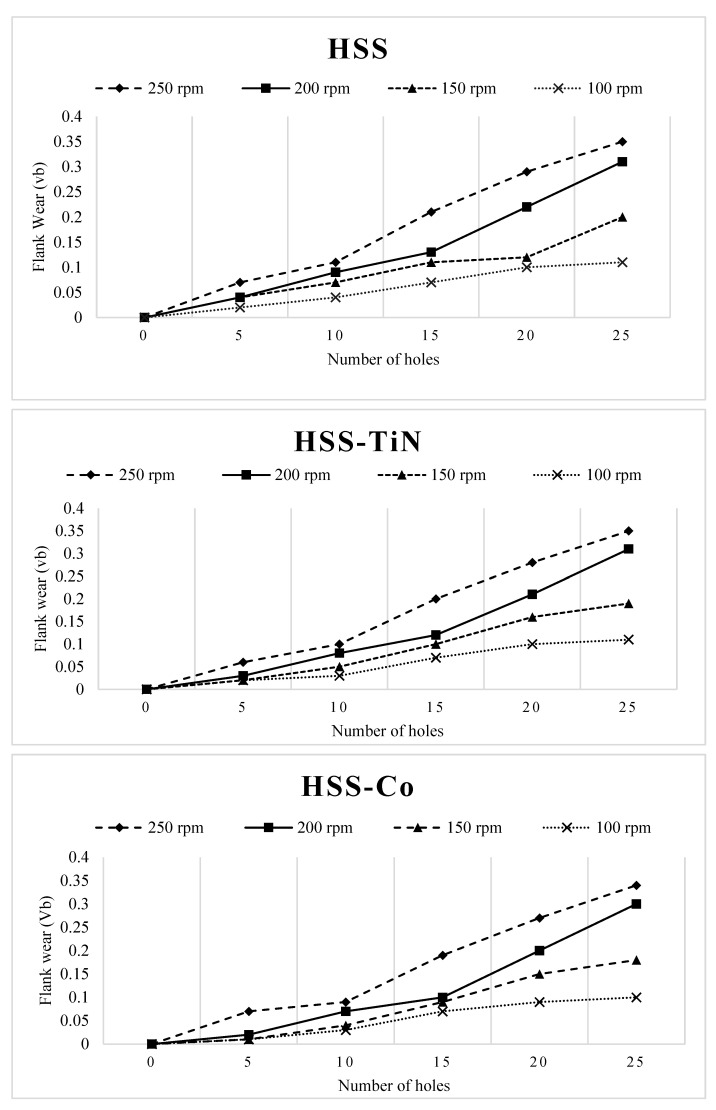
Comparison of flank wear of HSS, HSS-TiN, and HSS-Co at various spindle speeds.

Figure 7 shows the comparison of flank wear at various spindle speeds under the same cutting tool of HSS, HSS-TiN, and HSS-Co. In general, all trends of flank wear show that the rate of flank wear is faster with respect to the speed of the drilling processes. For the HSS tool, the maximum tool wear (Vb_max_) is already seen to exceed 100 rpm on the 20th hole, suggesting that the maximum of 20 holes can be drilled using HSS at 100 rpm. HSS can be used at faster spindle speed; somehow the Vb_max_ is exceeded at the 15th hole if 100 rpm is used. In addition, only 10 holes can be drilled on the materials if 200 rpm is applied. For the results of using the 250-rpm HSS tool, only five to six holes can be drilled before it exceeds Vb_max_. Remarkably, the HSS-TiN tool also does not show a significant improvement in drilling fly ash geopolymer. Similar to what was experienced on HSS uncoated tool, the use of 100 rpm on HSS-TiN shows the same number of holes as HSS when it reaches Vb_max_, i.e., 15 holes and 10 holes were recorded as the maximum number that can be drilled with 150 and 200 rpm, respectively. Interestingly, the coating of TiN helps the tool to stay a little bit longer in cutting at 250 rpm when the Vb_max_ at 250 rpm was recorded at the 10th hole.

For the HSS-Co cutting tool, it took the same 25 holes and 15 holes to reach Vb_max_ at 100 and 150 rpm, respectively. Nevertheless, the performance of HSS-Co helped the increase at 200 rpm. The Vb_max_ was recorded at the same 15th hole when 200 rpm was applied to the drilling process. Similarly, only 10 holes can be drilled using HSS-Co with 250 rpm before it reaches Vb_max_.

### 3.2. Surface Roughness Evaluation

The surface roughness of the material is one of the most important aspects that need to be considered when carrying a drilling process. The result of the surface roughness help to determine whether the selected parameter of machining is sufficient enough to create a good finishing quality to the product. Thus, maintaining a low value of surface roughness will produce a good finishing to the product itself within the demand and thus will create an added value to the production.

**Figure 8:**
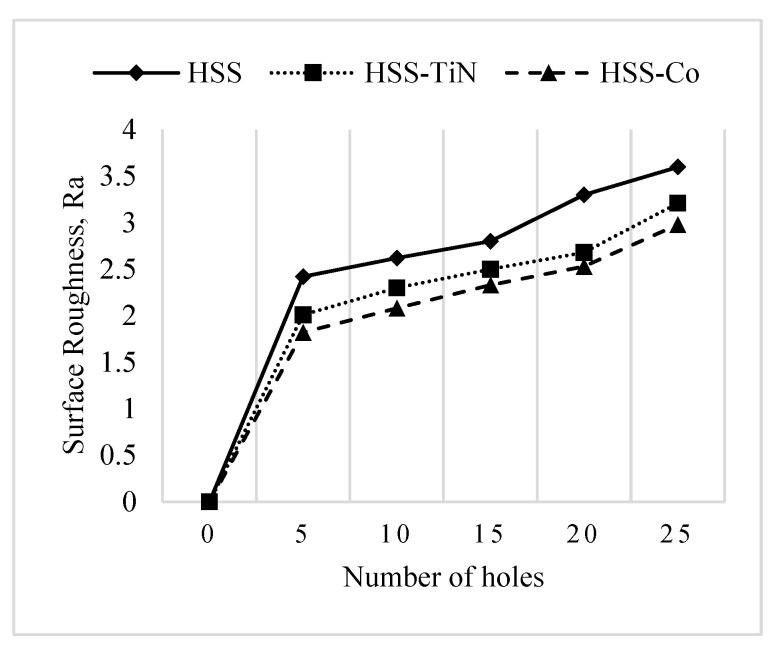
Surface roughness value of different cutting tools at a spindle speed of 250 rpm.

Figure 8 shows the comparison of surface roughness value for the cutting tools at a spindle speed of 250 rpm. The general trend shows gradual increases on Ra values indicating that the surface roughness quality is significantly affected by the engagement of the cutting tool with the workpiece. Another interesting point derived from the figure is that HSS-Co produces the lowest value of surface roughness, compared to HSS-TiN and uncoated HSS. When using uncoated drill tools, the thermal softening effects affected the edge of tools, resulting in damage. This situation could contribute to the high surface roughness of the drilled surface [12]. The HSS-Co-coated drill tool has a superior abrasive performance, which helps in shearing finer surface finish, compared to the other counterparts. During the drilling process, the cutting tool can retain hardness and stability at high impact force and high temperature, hence producing strong drilling action to slide and produce the holes consistently. It should be noted that the use of an uncoated drilling tool gave very high surface roughness, contributing to the major change in the percentage distribution. Therefore, for the best drilling performance, HSS-Co is suggested to be used along with a spindle speed of 100 rpm.

**Figure 9:**
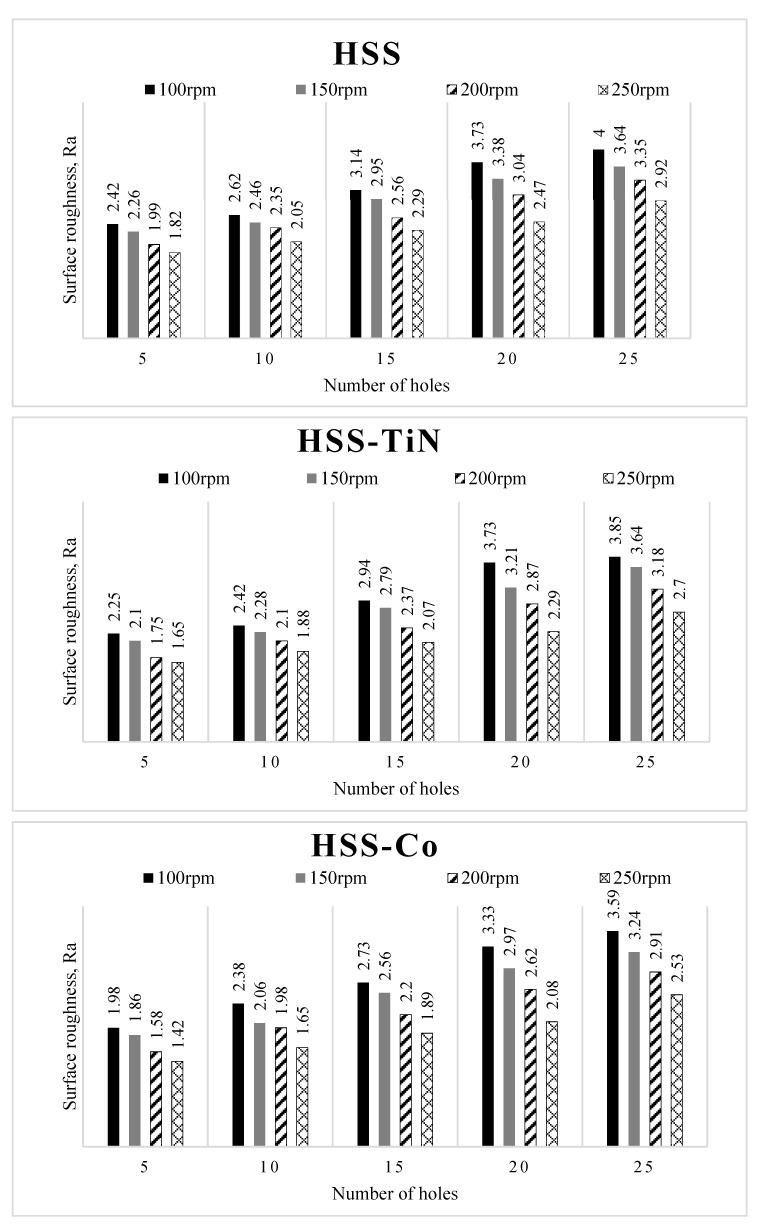
Surface roughness value of the cutting tools.

On the other hand, Figure 9 shows the comparison of surface roughness value of the cutting tools after drilling geopolymer at various spindle speeds. It can be seen from Figures that all the cutting tools showed exact behavior, in which the surface roughness values decrease with the increase in spindle speed from 100 rpm up to 250 rpm. The effects of spindle speed on the surface roughness obtained in this study are in agreement with the results of previous studies [30,31,32]. Although increasing the spindle speed may increase the quality of surface finish, higher spindle speed also comes with higher heat generated and therefore causes the tool to wear or blunt [32].

The results shown in Figure 9 show that the performance of surface roughness is closely related to the spindle speed. Increasing the spindle speed will lead to high friction, resulting in an increase in the temperature at the contact region. Hence, this will result in thermal softening of the workpiece material and thus reduce the thrust force and enable the machine to feed the drill with less energy, resulting in a better surface finish [32].

## 4. Conclusions

The first conclusion made is that fly ash geopolymer is machinable using drilling process either HSS, HSS-TiN, and HSS-Co drill bits, as indicated by the experiment. Even so, the experiment showed that almost all tools surpass the 0.10-mm targeted tool life limit after drilling 15 holes. Furthermore, all cutting tools show the behavior as expected, which is to wear gradually with respect to the number of drilled holes. On the other hand, the spindle speed is directly proportional with tool wear but inversely proportional with the surface roughness of the samples. The finding also shows HSS-TiN and HSS-Co increase the tool life at high spindle speed (200 rpm and above), compared to the HSS tool. HSS-Co exhibits the greatest tool life by showing the lowest value of flank wear and produces a better surface finish by exhibiting a low Ra value. Therefore, for the best drilling performance, Hss-Co is the most recommended tool to be used in drilling fly ash geopolymer material with a spindle speed of 100 rpm.

## Figures and Tables

**Table 1:** List of required tools in making geopolymer.

Tools	Description
Raw Material	Fly Ash class C
Activators	Sodium hydroxide, sodium silicate
Apparatus/Equipment	Wellbeing glasses, clean paper tissue, expendable nitrile gloves, laboratory garment, and 100 mm × 100 mm × 100 mm cubic mould.

**Table 2:** Parameter details in drilling fly ash geopolymer.

Parameter		Details	
Spindle speed (RPM)		250, 200, 150 and 100 RPM	
Feed rate (f) (mm/rev)		0.075 mm/rev	
Depth of cut (mm)		0.30 mm	
Cutting tool types		Coatings Thickness	Friction Coefficient
HSS (Danyang Daming Tools, Ltd, Danyang, China)		N/A	0.1–0.23
HSS Coated with TiN (Jiangsu Industry Co. Ltd., Yancheng, China)		6 µm	0.050–0.065
M35 HSS Co (5%) (Jiangsu Industry Co. Ltd., Yancheng, China)		N/A	0.030–0.045
Tool geometry specifications	No. of flute	2	
	Flute length (mm)	50	
	Shank diameter (mm)	6	
	Tool diameter (mm)	6	
	Overall length (mm)	102	
	Point angle (°)	135	
	Helix angle (°)	25	

**Table 3:** Tool wear effects on different spindle speeds using HSS, HSS-TiN, and HSS-Co.

No. of Holes	Tool Wear (mm)											
	Spindle Speed 250 rpm			Spindle Speed: 200 rpm			Spindle Speed: 150 rpm			Spindle Speed: 100 rpm		
	HSS	HSS-TiN	HSS-Co	HSS	HSS-TiN	HSS-Co	HSS	HSS-TiN	HSS-Co	HSS	HSS-TiN	HSS-Co
5	0.08	0.06	0.05	0.04	0.03	0.02	0.04	0.02	0.01	0.02	0.02	0.01
10	0.11	0.10	0.09	0.09	0.08	0.07	0.07	0.05	0.04	0.04	0.03	0.03
15	0.21	0.20	0.19	0.14	0.12	0.10	0.11	0.10	0.09	0.08	0.07	0.07
20	0.30	0.28	0.27	0.22	0.21	0.20	0.12	0.16	0.15	0.11	0.10	0.09
25	0.35	0.34	0.32	0.33	0.31	0.30	0.22	0.20	0.18	0.12	0.11	0.11

**Table 4:** Images of flank wears gradually emerge on HSS, HSS-TiN, and HSS-Co under a microscope magnification scale from 30× to150×.

Tools	Cutting Speed (rpm)	Initial Condition	Final Condition (25th Hole)
HSS	250	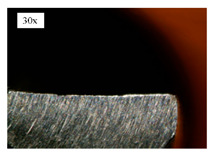	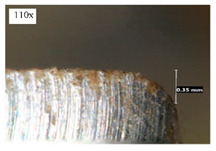
	100	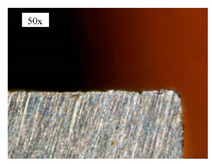	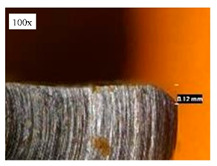
HSS-TiN	250	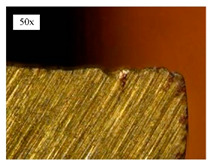	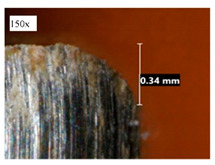
	100	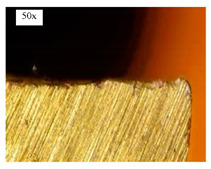	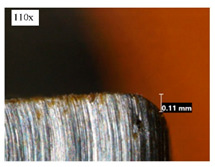
HSS-Co	250	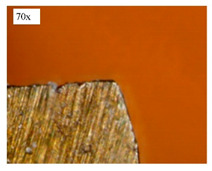	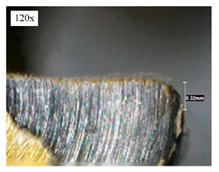
	100	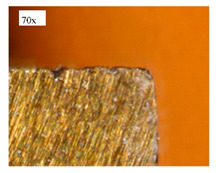	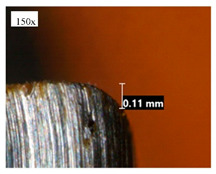

## Data Availability

The data presented in this study are available on request from the corresponding.

## References

[1] Lee W.H., Lin K.L., Chang T.H., Ding Y.C., Cheng T.W. (2020). Sustainable development and performance evaluation of mar-ble-waste-based geopolymer concrete. Polymers.

[2] Ahdaya M., Imqam A. (2019). Investigating geopolymer cement performance in presence of water-based drilling fluid. J. Pet. Sci. Eng..

[3] Liu X., Aughenbaugh K., Nair S., Shuck M., van Oort E. Solidification of Synthetic-Based Drilling Mud Using Geopolymers. Proceedings of the SPE Deepwater Drilling and Completions Conference.

[4] Colangelo F., Roviello G., Ricciotti L., Ferrándiz-Mas V., Messina F., Ferone C., Tarallo O., Cioffi R., Cheeseman C. (2018). Mechanical and thermal properties of lightweight geopolymer composites. Cem. Concr. Compos..

[5] Ghasemi A.H., Khorasani A.M., Gibson I. (2018). Investigation on the Effect of a Pre-Center Drill Hole and Tool Material on Thrust Force, Surface Roughness, and Cylindricity in the Drilling of Al7075. Materials.

[6] Madani H., Ramezanianpour A., Shahbazinia M., Ahmadi E. (2020). Geopolymer bricks made from less active waste materials. Constr. Build. Mater..

[7] Ma C.-K., Awang A.Z., Omar W. (2018). Structural and material performance of geopolymer concrete: A review. Constr. Build. Mater..

[8] Ranjitham M., Vishvapriya K., Pavya L.U.V. (2021). Experimental Investigation on Geopolymer Bricks. Advances in Materials Research.

[9] Hegab H., Darras B., Kishawy H. (2018). Towards sustainability assessment of machining processes. J. Clean. Prod..

[10] Aamir M., Tolouei-Rad M., Vafadar A., Raja M.N.A., Giasin K. (2020). Performance Analysis of Multi-Spindle Drilling of Al2024 with TiN and TiCN Coated Drills Using Experimental and Artificial Neural Networks Technique. Appl. Sci..

[11] Wang Q., Wang F., Zhang C., Chen C. (2019). Combined effects of various materials on tool wear in drilling of Ti/CFRP stacks. Proc. Inst. Mech. Eng. Part C J. Mech. Eng. Sci..

[12] Xu J., Zhou L., Chen M., Ren F. (2019). Experimental study on mechanical drilling of carbon/epoxy composite-Ti6Al4V stacks. Mater. Manuf. Process..

[13] Geng D., Liu Y., Shao Z., Lu Z., Cai J., Li X., Jiang X., Zhang D. (2019). Delamination formation, evaluation and suppression during drilling of composite laminates: A review. Compos. Struct..

[14] Bhat R., Mohan N., Sharma S., Shandilya M., Jayachandran K. (2019). An integrated approach of CCD-TOPSIS-RSM for op-timizing the marine grade GFRP drilling process parameters. Mater. Today.

[15] Tabatabaeian A., Baraheni M., Amini S., Ghasemi A.R. (2019). Environmental, mechanical and materialistic effects on delami-nation damage of glass fiber composites: Analysis and optimization. J. Compos. Mater..

[16] Paizun A.A., Fathullah M., Abdullah M.M.A., Shayfull Z., Tahir F. (2019). Surface roughness optimization on rubberized fly ash geopolymer in lathe operation using Taguchi method. AIP Conf. Proc..

[17] Yu T.C., Fathullah M., Abdullah M.M.A., Shayfull Z., Tahir F. (2019). Tool wear evaluation on rubberized fly ash geopolymer milling. AIP Conf. Proc..

[18] Norsyafikah S., Fathullah M., Abdullah M.M.A., Shayfull Z., Tahir F. (2019). Surface integrity of rubberized geopolymer fly ash geopolymer in milling machining. AIP Conf. Proc..

[19] Kamilah N., Fathullah M., Abdullah M.M.A., Faris M.A., Tahir F., Shayfull Z., Nasir S.M., Shazzuan M., Wazien A.Z.W. (2002). Surface integrity of steel fibre reinforced fly ash geopolymer in CNC lathe operation. AIP Conf. Proc..

[20] Harris M., Qureshi M.A., Saleem M.Q., Khan S.A., Bhutta M.M. (2017). Carbon fiber-reinforced poly composite drilling via aluminum chromium nitride-coated tools: Hole quality and tool wear assessment. J. Reinf. Plast. Compos..

[21] Michael G. (2017). Optimization of drilling parameters on CFRP composites. Int. J. Sci. Dev..

[22] Aamir M., Tolouei-Rad M., Giasin K., Nosrati A. (2019). Recent advances in drilling of carbon fiber–reinforced polymers for aerospace applications: A review. Int. J. Adv. Manuf. Technol..

[23] Wei Y., An Q., Ming W., Chen M. (2016). Effect of drilling parameters and tool geometry on drilling performance in drilling carbon fiber–reinforced plastic/titanium alloy stacks. Adv. Mech. Eng..

[24] Rao Y.S., Mohan N.S., Shetty N., Shivamurthy B. (2019). Drilling and structural property study of multi-layered fiber and fabric reinforced polymer composite—A review. Mater. Manuf. Process..

[25] Ali M., Xiang L., Yue D., Liu G. (2018). Assessment of cutting performance of cemented tungsten carbide drills in drilling mul-tidirectional T700 CFRP plate. J. Manuf. Mater. Proc..

[26] Ucun I., Kaplan S. (2017). Determination of tool wear and chip formation in drilling process of AISI 1045 material using plasma-nitrided high-speed steel drill bits. Proc. Inst. Mech. Eng. B J. Eng. Manuf..

[27] Shetty N., Herbert M.A., Shetty D.S., Vijay G.S., Shetty R.S.B. (2015). Experimental investigation in drilling of carbon fiber reinforced polymer composite using HSS and solid carbide drills. Int. J. Curr. Eng. Technol..

[28] Aamir M., Tu S., Tolouei-Rad M., Giasin K., Vafadar A. (2020). Optimization and Modeling of Process Parameters in Multi-Hole Simultaneous Drilling Using Taguchi Method and Fuzzy Logic Approach. Materials.

[29] Iqbal M., Bahri S., Akram A. (2019). Effect of cutting parameter on tool wear of HSS tool in drilling of Kevlar composite panel. IOP Conf. Ser. Mater. Sci. Eng..

[30] Uddin M., Basak A., Pramanik A., Singh S., Krolczyk G.M., Prakash C. (2018). Evaluating Hole Quality in Drilling of Al 6061 Alloys. Materials.

[31] Kumar K., Zindani D., Davim J.P. (2018). Advanced Machining and Manufacturing Processes.

[32] Bhushan R.K., Kumar S., Das S. (2010). Effect of machining parameters on surface roughness and tool wear for 7075 Al alloy SiC composite. Int. J. Adv. Manuf. Technol..

